# Evidence and reporting standards in N-of-1 medical studies: a systematic review

**DOI:** 10.1038/s41398-023-02562-8

**Published:** 2023-07-18

**Authors:** Prathiba Natesan Batley, Erica B. McClure, Brandy Brewer, Ateka A. Contractor, Nicholas John Batley, Larry Vernon Hedges, Stephanie Chin

**Affiliations:** 1 University of Louisville, Louisville, KY USA; 2 Purdue University, West Lafayette, IN 47907 USA; 3 University of North Texas, Denton, TX USA; 4 Batley Consulting LLC, Louisville, KY USA; 5 Northwestern University, Evanston, IL USA

**Keywords:** Scientific community, Human behaviour

## Abstract

N-of-1 trials, a special case of Single Case Experimental Designs (SCEDs), are prominent in clinical medical research and specifically psychiatry due to the growing significance of precision/personalized medicine. It is imperative that these clinical trials be conducted, and their data analyzed, using the highest standards to guard against threats to validity. This systematic review examined publications of medical N-of-1 trials to examine whether they meet (a) the evidence standards and (b) the criteria for demonstrating evidence of a relation between an independent and an outcome variable per the What Works Clearinghouse (WWC) standards for SCEDs. We also examined the appropriateness of the data analytic techniques in the special context of N-of-1 designs. We searched for empirical journal articles that used N-of-1 design and published between 2013 and 2022 in PubMed and Web of Science. Protocols or methodological papers and studies that did not manipulate a medical condition were excluded. We reviewed 115 articles; 4 (3.48%) articles met all WWC evidence standards. Most (99.1%) failed to report an appropriate design-comparable effect size; neither did they report a confidence/credible interval, and 47.9% reported neither the raw data rendering meta-analysis impossible. Most (83.8%) ignored autocorrelation and did not meet distributional assumptions (65.8%). These methodological problems could lead to significantly inaccurate effect sizes. It is necessary to implement stricter guidelines for the clinical conduct and analyses of medical N-of-1 trials. Reporting neither raw data nor design-comparable effect sizes renders meta-analysis impossible and is antithetical to the spirit of open science.

## Introduction

N-of-1 studies, which are special cases of single case experimental designs (SCEDs), are important in the medical field, where treatment decisions may be made for an individual patient, or where large-scale trials are not always possible or even appropriate such as when treating rare diseases, comorbid conditions, or concurrent therapies [1]. In fact, n-of-1 trials have been suggested as a valuable scientific method in precision medicine [2] and are particularly important in the field of psychiatry. Recently, the British Journal of Psychiatry published a special issue focusing on precision medicine and personalized healthcare in psychiatry. The What Works Clearinghouse (WWC [3, 4]) standards for SCEDs noted several requirements to increase rigor pertaining to evidence standards and demonstration of treatment effect between the independent and the outcome variable. It is important to note here that the term outcome variable refers to the dependent variable and not a medical outcome such as morbidity, mortality, etc. The purpose of these standards is to address validity concerns in SCEDs. What is unclear is if these important standards have been adopted in medical research. To this end, we conducted a systematic literature review using the PRISMA (Preferred Reporting Items for Systematic Literature Reviews and Meta-analyses) guidelines to address the following aims:

1. To examine whether N-of-1 trials meet WWC evidence standards; namely, independent variables being systematically manipulated, outcome variables measured systematically over time by more than one assessor, interobserver agreement data being collected in each phase for at least 20% of data points per condition, including 3 or more attempts to demonstrate a treatment effect at three different points in time, and having the number of required data points per case/phase,
2. To examine whether evidence of a treatment effect is examined in N-of-1 trials per WWC standards (namely, immediacy, consistency, changes in level/trend, and effect sizes), and
3. To examine the data and methodological characteristics of the studies such as phase lengths, inclusion of autocorrelation, appropriateness of the type of analysis for the data type, sensitivity, and subgroup analyses.

Although the SPENT (SPIRIT extension for N-of-1 trials) checklist [5] has been developed specifically for n-of-1 protocols, we chose the WWC standards because the former focuses on improving the completeness and transparency of N-of-1 protocols, whereas the latter focuses on addressing threats to validity and reporting guidelines to establish evidence of treatment effect between the independent and the outcome variable. Therefore, the latter speaks more to the validity aspects of N-of-1 trials.

## Methods

### Literature search

#### Inclusion/exclusion criteria

The review followed the 2020 PRISMA recommendations [6] (Supplementary Table 1) and guidelines from the Cochrane Collaboration for data extraction and synthesis [7]. Included studies were peer-reviewed, published in medical journals, examined medical outcomes, used SCED/N-of-1, were empirical articles, and in the English language. Only medical conditions listed in International Classification of Diseases (ICD-10) [8] or Diagnostic and Statistical Manual of Mental Disorders (DSM-5) [9] were included in the present study to retain a meaningful scope and align with widely used clinical practices. Online Supplementary Table 1 gives the PRISMA checklist and how they were met for the current study.

#### Search strategy

The following databases were searched: PubMed and Web of Science. These databases were chosen because these search engines have reproducible search results in different locations and at different times. Exact search terms were: "n-of-1*" OR "N-of-1 trial" OR "N-of-1 design" OR "single case design" OR “single subject design” OR “single case experimental design” AND “drug” OR “therapy” OR “intervention” OR “treatment” in the title, abstract, or keywords. The dates of publication were restricted to between January 1, 2013 and May 3, 2022 for relevance, sufficiency, and feasibility as the WWC Standards for SCEDs were published in 2010 and later in 2013. The search ended on May 3, 2022.

#### Data management

References and abstracts of articles found from the initial search were downloaded into the reference management software EndNote. Duplicate reference entries were removed. The remaining reference entries were transported to a Google Sheets file by and for independent review by two co-authors (EM and BB) for inclusion criteria. Reliability of electronic search results was established through replication of the electronic search and an inter-rater comparison of the number of identified articles (100% agreement).

#### Selection process

Two co-authors independently (EM and BB) screened 341 articles (title and article abstract review) to determine eligibility of articles for the current review. From this initial screening, 189 articles were identified as potentially eligible and were subjected to a second screening. The two co-authors then independently reviewed the 189 articles (full text) to ensure their eligibility for this review. Articles that were not empirical work (e.g., protocol, commentary), and articles that were not N-of-1 trials or did not have a medical outcome variable were excluded independently leading to a total of 115 articles that met all inclusion criteria (100% agreement).

#### Coding process

There were 4 coders. Two were experts in statistical methodology and three were experts in SCEDs. One co-author (EM), as the primary coder, conducted data extraction from 115 eligible articles. To obtain inter-reliability estimates, 30 (26.09%) of the included articles were additionally coded by two other co-authors (BB and SC) through random assignment. Before coding the articles included in the review, researchers calibrated coding reliability by using the coding tool to analyze studies that did not meet the inclusion criteria. Interobserver agreements during calibration were measured at 94.3%. When discussing whether a specific study met an individual indicator, areas of incongruity were discussed until researchers reached consensus. Once reliability above 90% was established, researchers began coding the articles included in the review (coding tool available from first author). Interobserver agreement for all coded articles was measured at 93.1%. Finally, the first author (PNB) recoded all the articles to ensure 100% agreement between the first author and the coding of the other three co-authors.

#### Risk of bias assessment

as given by the Risk of Bias in Systematic Reviews Tool (ROBIS) (http://www.bristol.ac.uk/population-health-sciences/projects/robis/) is in Table 1. Additionally, the online Supplementary Table 2 gives the risk of bias in not meeting evidence standards, in reporting treatment effect, and in inappropriate data analysis for each study.

**Table 1:** Judging risk of bias in the systematic review.

Domain	Concern	Rationale for concern
Concerns regarding specification of study eligibility criteria	Language, years	We might have missed scientific studies published in other languages or older publications of import
Concerns regarding methods used to identify and/or select studies	Database search	It is possible that the two databases we searched in is not exhaustive of all medical literature, or the library did not have subscription to some of the journals
Concerns regarding methods used to collect data and appraise studies	Expertise	All the authors are experts in SCEDs but none are experts in medicine
Concerns regarding the synthesis and findings	None	

#### Rating evidence

**Fig. 1:**
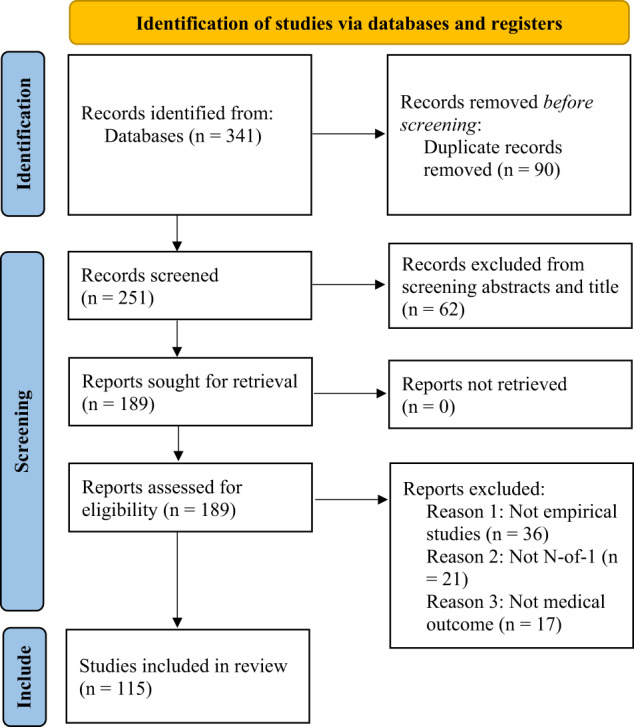
PRISMA 2020 flow diagram for the systematic review. The number of articles identified, screened, retrieved, assessed, and finally retained. *n* represents the sample size.

All studies were N-of-1 studies. According to Oxford center for evidence-based medicine (OCEBM, https://www.cebm.ox.ac.uk/files/levels-of-evidence/cebm-levels-of-evidence-2-1.pdf) all these studies will be level 3 studies because they manipulate the control arm of a randomized trial (Fig. 1).

#### Statistical analysis

Descriptive analysis such as frequency and percentages are reported. Table 2 outlines information on number of studies meeting the WWC [3, 4] requirements for meeting evidence standards. Table 3 outlines information on number of studies demonstrating how treatment effect was determined per WWC standards [3, 4] (immediacy, changes in level/trend, effect sizes/confidence or credible intervals, consistency, effect sizes), and different methodological characteristics (e.g., type of analysis conducted, whether this was appropriate for the data [if data met the assumptions of the analyses], and whether autocorrelations were included in the models). Additionally, we coded the characteristics of the study such as the number of phases, phase length, type of outcome variable, types of effect sizes, data distribution assumptions met, accounting for carryover effect, intraclass correlation, sensitivity analysis, and subgroup analysis.

**Table 2:** Summary of the 115 studies and whether and how they meet WWC evidence standards.

N-of-1 design identification	Identified	48	41.90%
	Not Identified	67	58.10%
Type of N-of-1 design	ABAB	37	32.50%
	MBD	15	12.80%
	ATD	16	13.70%
	CCD	4	3.40%
	Combination	0	0.00%
	AB	28	23.90%
	BA	1	0.90%
	ABA	10	9.40%
	BAB	1	0.90%
	ABC	3	2.60%
Evidence standards as described by the WWC	Independent variable was not systematically manipulated	1	0.90%
	Outcome variable was not measured systematically over time by more than one assessor	3	2.60%
	IOA was not collected in each phase for at least 20% of data points per condition	111	95.70%
	Study did not include ≥3 attempts to demonstrate a treatment effect at three different points in time	46	39.30%
	Study did not have the number of required data points (3–5) per case/phase	29	24.80%
	Study met evidence standards	4	4.30%

**Table 3:** Data and analytical reporting characteristics of the 115 studies.

Methodological characteristic	Minimum	Average	Maximum
Number of phases	1	4.31	62
Minimum duration	4 min	82.79 days	75 months
Number of participants	1	12.5	200
Autocorrelation magnitude	−0.925	NA^b^	2^a^
Mean difference	−8	NA^b^	100
**Methodological characteristic**	**Subtype**	**Frequency**	**Percentage**
Type of outcome variable	Count	35	31.60%
	Duration	1	0.90%
	Frequency	3	2.60%
	Rate	7	6.00%
	Qualitative	2	1.70%
	Rating Scale	63	53.80%
	Other	4	3.40%
Autocorrelation	Included	18	16.20%
	Not Included	97	83.80%
How was treatment effect determined?	Immediacy	7	6.00%
	Level Change	105	91.50%
	Slope Change	0	0.00%
	Effect Size	1	0.90%
	None	2	1.70%
Immediacy	Yes	7	6.00%
	No	108	94.00%
Level change	Yes	113	98.30%
	No	2	1.70%
Slope change	Yes	24	20.50%
	No	91	79.50%
Effect size	Yes	70	61.50%
	No	45	38.50%
Consistency	Yes	30	27.40%
	No	85	72.60%
Type of analysis	Multi-Level modeling	8	6.80%
	Regression modeling	5	4.30%
	Paired t-test	23	19.70%
	Mixed effects model	8	6.80%
	Meta-analysis	1	0.90%
	Analysis of variance (ANOVA)	7	6.00%
	Bayesian hierarchical modeling	7	6.00%
	Logistic regression	1	0.90%
	Linear modeling	6	6.00%
	Visual	22	19.70%
	Other	8	6.80%
	None	19	16.20%
Correct distributional assumptions	Yes	39	34.20%
	No	76	65.80%
Type of effect size reported	R	9	7.70%
	Cohen’s d	8	7.70%
	G	1	0.90%
	Δ	0	0.00%
	ϕ	1	0.90%
	None	45	38.50%
	Mean difference	52	44.40%
Were the entire data reported in a way that they could be replicated for further analysis (e.g., plots or raw data)?	Yes	59	52.10%
	No	56	47.90%
Did the study account for carryover effect (effect of the treatment into the next phase after removal of treatment)?	Yes	47	40.20%
	No	68	59.80%
Period effects	Yes	47	40.20%
	No	68	59.80%
Intra-subject correlation	Yes	17	15.40%
	No	98	84.60%
Confidence/credible interval for effect size	Yes	45	39.30%
	No	70	60.70%
Sensitivity analysis	Yes	14	12.80%
	No	101	87.20%
Subgroup analysis	Yes	13	11.10%
	No	102	88.90%

^a^This is not statistically possible.

^b^Means could not be computed due to missing information or articles providing ranges of the statistic.

## Results

As outlined in Table 2, of the 115 studies, 68 (59.13%) did not identify the type of SCED used. Therefore, we identified these designs. To answer research question 1 about how many studies passed all the WWC criteria: only 4 (3.48%) studies passed all the WWC criteria for meeting evidence standards (see online Supplementary Table 2). Specifically, IOA (interobserver agreement) was not collected in each phase for at least 20% of data points per condition for 95.7% of the cases. It is possible that sometimes this is not applicable when the outcome variable is measured using an instrument and not necessarily by observers. However, this was the case for only 3 (2.6%) of the studies. 39.3% of the studies did not include ≥ 3 attempts to demonstrate a treatment effect at three different points in time which is a threat to validity because at least 3 independent demonstrations of treatment effect are required to show that the treatment effect did not happen due to random variation in data. Demonstrating a treatment effect at least 3 times is important in N-of-1 studies because the question of whether the treatment effect is replicable across phases or cases is answered by this demonstration, which has obvious impact on validity. 24.8% of them did not have the number of required data points (3–5) per case/phase. This means that the studies were terribly underpowered. It is impossible to obtain reliable estimates of phase means or worse yet, determine if the treatment effect varied with time.

Regarding research question 2, as outlined in Table 3, most studies (98.3%) determined change in level between phases to report evidence of treatment effects. Consistency was not investigated by 72.6% of the studies and 38.5% of the studies did not report any effect size. The most reported effect size was an unstandardized mean difference between the phases which ranged from −8 to 100. Further, 60.7% of the studies did not report a confidence/credible interval estimate for effect size. The issue with simply reporting an unstandardized mean difference effect size is that there are no units to understand the metric of the effect size. For instance, an unstandardized mean difference of 3 units would be a significant drop in hemoglobin A1C versus a trivial 3 unit drop in systolic blood pressure.

Regarding question 3, only 6% of the studies determined immediacy which is a requirement for causal evidence in SCEDs. Immediacy informs the researcher as to how immediately a treatment took effect, so it eliminates any other extraneous reason for a change in the outcome variable. Therefore, in the absence of a substantive reason for delay in the treatment influencing the outcome variable, immediacy is paramount. Autocorrelation was not modeled in 83.8% of the studies. Of these, one study reported a statistically impossible autocorrelation value of 2. When not including autocorrelations for autocorrelated data, we are assuming the data are independent of each other and any parametric analysis that is employed would be used on data that violate the basic independence of observation assumption. This could lead to wildly inaccurate estimates. We coded the analysis as not being appropriate for the data if the data type did not meet the distributional assumptions of the type of analysis being conducted (65.8%). Again, this could lead to inaccurate estimates. Meta-analysis can be conducted when authors provide a reliable design-comparable effect size estimate or report the complete dataset which is common practice in SCEDs using a data plot. Only one study (0.9%) included autocorrelation and corrected for small sample size in their computations by reporting a design-comparable effect size, i.e., Hedges’ g [10, 11]. 47.9% of the studies did not report raw data to be considered for future meta-analysis. Although several studies included more than one participant, only 15.4% computed and reported intraclass correlation. Intraclass correlation is necessary to be computed because it is the correlation among the scores within the individuals or the ratio of between cluster variance (i.e., the variability between people) to the total variance. That way we know how much of the variance in the outcome variable is due to differences between people and within people. This is also necessary to compute the appropriate effect size.

## Discussion

It is highly concerning that only 4 of the 115 (3.48%) studies met the WWC evidence standards. While it has become the default that the presence or absence of phenomena be accompanied by a measure of its magnitude, it is still unfortunate that this essential practice is not being upheld universally. The most reported mean difference effect size is not scale free and therefore, is difficult to interpret and aggregate across studies in meta-analysis. Other effect sizes were Cohen’s d, rate ratio, incidence ratio, etc. which did not include autocorrelation or correction for small sample size in their computations. These are also not design-comparable because they are within-subject effect sizes that are not computed across participants. Regarding immediacy, there are models developed specifically to determine immediacy and its magnitude that can help strengthen the evidence of effective treatments [12, 13]. It would behoove medical N-of-1 researchers to examine these methodologies.

Not including autocorrelation in the statistical model is problematic because we know that SCED data are autocorrelated and not modeling autocorrelation leads to erroneous estimates of effect and inflated Type-I error rates [14–19]. Estimating autocorrelations with sufficient accuracy for shorter time-series is still in its fledgling stage, but it certainly cannot be ignored. We should also remember that most SCEDs have shorter time-series and/or fewer individuals which implies that violation of distributional assumptions becomes more serious, and results are more erroneous when these are ignored. This drawback is exacerbated by ignoring autocorrelations. Reporting intraclass correlation is important for understanding how similar the subjects were to each other and reporting adequate information such as the raw data or a design-comparable effect sizes is essential for scientific progress through meta-synthesis.

Given the findings, here are our recommendations for policymakers, gatekeepers of research standards, and editors of journals that are interested in ensuring the most robust level of conduct and analysis of N-of-1 trials.

1. Discuss the implications and set standards based on guidelines for best practices founded not just on research conduct in one discipline, but on interdisciplinary research conduct. Specifically, the social sciences and education/psychology research analyses of SCEDs are conducted based on the standards set forth by the WWC. The SPENT guidelines [5] focus on completeness and transparency of N-of-1 protocols. However, there is a need to derive the best practices based on both and combine the wealth of knowledge created in both fields.
2. WWC lays specific standards for meeting evidence standards and reporting treatment effect because the data are shorter time-series, are autocorrelated, and often have few participants. These must be strictly adhered to, especially in medical research which has high stakes impact for the subjects.
3. Use common terminology to facilitate interdisciplinary research. A classic example is identifying the type of SCED. This would allow us to set clear expectations for conduct of research following the highest standards.
4. Support more methodological contributions in medical journals particularly with respect to analyzing N-of-1 data.
5. Emphasize the imperative nature of reporting effect sizes and confidence/credible intervals when statistically analyzing data; in fact, make this a requirement.
6. Report enough information to facilitate meta-analysis, for that is the ultimate aim of most research. This can be either in the form of raw data or design-comparable effect sizes.
7. Encourage investigation of the impact of autocorrelations in estimating effect sizes. Require the inclusion of estimating and reporting autocorrelations.
8. Support more development of innovative and easy-to-use tools for analysis of N-of-1 analyses.

The limitations include including only English language articles, potentially excluding articles that might not have been part of the databases we searched in, human errors in coding (although we had 4 independent coders) and excluding N-of-1 studies that did not use our search terms in their title, abstract, or keywords.

In sum, N-of-1 studies in the medical field are not currently adhering to important standards that guard validity both in their conduct and in their analysis. Neither do they report adequate information to facilitate meta-analytic work. Editorial and research standards must require increased rigor in this experimental design which is still in its nascent stage.

## Supplementary information

Supplementary Table 1

Supplementary Table 2

## Data Availability

All data used in this article are made available through online supplemental Table 2. This also contains all the variables used for coding and the references.
